# Cost-utility analysis of adjuvant imatinib treatment in patients with high risk of recurrence after gastrointestinal stromal tumour (GIST) resection in Thailand

**DOI:** 10.1186/s12962-018-0169-9

**Published:** 2019-01-08

**Authors:** Thanaporn Bussabawalai, Kittiphong Thiboonboon, Yot Teerawattananon

**Affiliations:** 1 Health Intervention and Technology Assessment Program (HITAP), Department of Health 6th floor, 6th Building, Ministry of Public Health, Tiwanon Road, Muang, Nonthaburi, 11000 Thailand; 2 Present Address: Centre for Health Economics Research and Evaluation, University of Technology Sydney, Haymarket, Sydney, Australia

**Keywords:** Cost-utility analysis, Adjuvant imatinib, Recurrence, Gastrointestinal stromal tumour, GIST

## Abstract

**Background:**

Many patients develop tumour recurrence within a few years after undergoing surgical resection of gastrointestinal stromal tumours (GIST). Adjuvant imatinib treatment is recommended for patients with high risk of GIST recurrence as it can improve recurrence-free survival and overall survival of patients. This study aims to assess the cost-utility of adjuvant imatinib in patients with high risk of GIST recurrence after surgery compared with no adjuvant therapy in Thailand.

**Methods:**

A Markov model was developed to estimate lifetime costs and outcomes of using adjuvant imatinib treatment and other treatment alternatives if recurrence occurred compared with the current situation of no adjuvant therapy in high-risk patients after surgery. A 1-month cycle length was deployed in the model. Transition probabilities were derived from literature review. Costs were collected and calculated for the year 2014 from a societal perspective. Future costs and outcomes were discounted at 3% per year. One-way and probabilistic sensitivity analyses were conducted to assess parameter uncertainties.

**Results:**

Three years of adjuvant imatinib treatment followed by imatinib treatment and best supportive care if recurrence occurred after or during adjuvant therapy, respectively, was the best option as it produced more health outcomes (1.23 life years (LYs) and 1.16 quality-adjusted life years (QALYs)) compared to no adjuvant therapy while yielding the lowest incremental cost-effectiveness ratio (ICER) of 1,648,801 Thai Baht (THB) per QALY gained. Three years of adjuvant imatinib treatment followed by sunitinib treatment if recurrence occurred had an ICER of 2,608,264 THB per QALY gained compared to the best option, while other options were dominated. A one-way sensitivity analysis showed that the utility of patients receiving adjuvant imatinib had the greatest effect on the model, followed by the discount rate and probability of GIST recurrence.

**Conclusions:**

Adjuvant imatinib treatment improved the health benefits of patients with high risk of GIST recurrence. However, in the Thai context, it was not cost-effective at the current price.

## Background

Gastrointestinal stromal tumours (GIST) are the most common mesenchymal tumours of the gastrointestinal (GI) tract [1]. GIST can be found in any part of the GI tract but mostly occur in the stomach and small intestine. Most cases of GIST are associated with mutations in either KIT or PDGFRA (platelet-derived growth factor receptor alpha). The activation of those mutations leads to the end result of an increase in cellular proliferation and a decrease in apoptosis [1–3]. GIST can be diagnosed by histological examinations, CD117 (c-kit) immunohistochemistry, and other imaging techniques such as computed tomography (CT), magnetic resonance imaging (MRI), upper GI endoscopy, and fluorine-18-fluorodeoxyglucose positron emission tomography (FDG-PET). The clinical presentation of GIST can be asymptomatic or symptomatic; common symptoms of GIST include abdominal pain, and GI bleeding and obstruction [2, 4].

The standard treatment for localized GIST is a complete resection [4]; however, many patients have disease recurrence after surgery [5]. The evidence from a randomized phase III, placebo-controlled, multicenter trial showed that 400 mg of tyrosine kinase inhibitor, imatinib mesylate per day for 1 year significantly improved recurrence-free survival (RFS) compared with the placebo (hazard ratio 0.35; 95% CI 0.22–0.53; p < 0.0001) in patients with complete resection of GIST greater than 3 cm [6]. In addition, the results from another randomized phase III study comparing the efficacy of 1 and 3 years of adjuvant imatinib treatment in patients with high risk of GIST recurrence after surgery indicated that extending the duration of adjuvant imatinib treatment to 3 years could improve both RFS (hazard ratio 0.46; 95% CI 0.32–0.65; p < 0.001) and overall survival (hazard ratio 0.45; 95% CI 0.22–0.89; p = 0.02) compared to just 1 year of adjuvant imatinib treatment [7].

According to the benefits of adjuvant imatinib treatment based on available evidence, some agencies recommend adjuvant imatinib therapy. For example, the National Comprehensive Cancer Network (NCCN) in the United States recommends adjuvant imatinib treatment after resection for primary GIST for at least 12 months in intermediate- to high-risk patients [8], whereas the European Society for Medical Oncology (ESMO) in Europe recommends adjuvant therapy for 3 years only in patients with high risk of recurrence (it is not recommended for patients with low risk of recurrence) [9]. There are several risk stratification schemes available to determine the risk of GIST recurrence after surgery such as the National Institutes of Health (NIH) consensus criteria, Armed Forces Institute of Pathology (AFIP) criteria, and Modified NIH criteria. Many prognostic factors are considered including tumour size, mitosis count, tumour site, and tumour rupture, depending on the criteria of each scheme [9, 10]. However, experts in Thailand normally estimate the risk of GIST recurrence based on the Modified NIH criteria. This scheme takes all four of those previously mentioned factors into consideration, whereas the NIH consensus criteria focus only on tumour size and mitosis count. Patients with a large tumour and/or frequent mitoses have a high risk of recurrence. Non-gastric sites also increase the risk of recurrence, according to the Modified NIH criteria. Additionally, tumour rupture also leads to a high risk of recurrence regardless of sizes, sites, and mitosis count [10]. There is no report on the incidence and prevalence of GIST in Thailand. However, experts estimate that the new cases of GIST patients who undergo resections and have a high risk of recurrence are approximately 100 persons per year in Thailand.

In Thailand, 400 mg of imatinib per day is included in the National List of Essential Medicines (NLEM) only for the treatment of patients with unresectable and/or metastatic GIST, whereas adjuvant imatinib treatment for patients with high risk of GIST recurrence after surgery has not yet been included. An economic evaluation is needed to inform the Subcommittee for the Development of the NLEM about the cost-effectiveness of this new indication of imatinib treatment to determine whether to include it into the NLEM or not. Therefore, this study aims to assess the cost-utility of adjuvant imatinib therapy in patients with high risk of GIST recurrence after surgery compared with no adjuvant therapy in Thailand.

## Methods

A Markov model was developed to estimate the costs and health outcomes based on a societal perspective of using adjuvant imatinib treatment and drug treatment alternatives if recurrence occurred, compared with the current situation of no adjuvant therapy. The study population was primary localized GIST patients who underwent complete resections and had a high risk of recurrence as determined by the Modified NIH criteria. A lifetime horizon was performed with a 1-month cycle length in the model. As the time horizon of this study was more than 1 year, future costs and outcomes were discounted at 3% per year.

According to national and international practice guidelines—as well as being in line with the criteria of using imatinib in the Thai NLEM that allows for the use of 400 mg of imatinib per day only for metastatic GIST—this study focused on four alternative treatment options varying in accordance with adjuvant treatment and treatment after disease recurrence and progression. The details of each option are described (Table 1) as follows:

Option 1: No adjuvant imatinib therapy after surgery was given. If disease recurrence occurred, patients received 400 mg of imatinib per day. If the disease continued to progress, patients stopped imatinib treatment and received best supportive care (baseline case).
Option 2: 400 mg of adjuvant imatinib per day after surgery was given for 1 year (option 2.1) or 3 years (option 2.2).
  - 2.1: If disease recurrence occurred during adjuvant therapy, patients stopped adjuvant imatinib treatment and received best supportive care.
  - 2.2: If disease recurrence occurred after adjuvant therapy had been completed, patients received 400 mg of imatinib per day. If the disease continued to progress, patients stopped imatinib treatment and received best supportive care.
Option 3: 400 mg of adjuvant imatinib per day after surgery was given for 1 year (option 3.1) or 3 years (option 3.2) .
  - 3.1: If disease recurrence occurred during adjuvant therapy, patients stopped adjuvant imatinib treatment and received a treatment of 50 mg of sunitinib per day. If the disease continued to progress, patients received best supportive care instead of the sunitinib treatment.
  - 3.2: If disease recurrence occurred after adjuvant therapy had been completed, patients received 400 mg of imatinib per day. If the disease continued to progress, patients stopped the imatinib treatment and received a treatment of 50 mg of sunitinib per day. If the disease still continued to progress, patients received best supportive care instead of the sunitinib treatment.
Option 4: no adjuvant imatinib therapy after surgery was given. If disease recurrence occurred, patients received 400 mg of imatinib per day. If the disease continued to progress, patients stopped imatinib treatment and received a treatment of 50 mg of sunitinib per day. If the disease still continued to progress, patients received best supportive care instead of the sunitinib treatment.

**Table 1:** Alternative treatment options

Options	Adjuvant imatinib treatment	Treatment after recurrence occurred	
		Recurrence	Progression
Option 1 (baseline case)	No	Imatinib	BSC
Option 2.1 Option 2.2	1 year 3 years	Imatinib (recurrence occurred after adjuvant therapy completed)	BSC
		BSC (recurrence occurred during adjuvant therapy)	
Option 3.1 Option 3.2	1 year 3 years	Imatinib (recurrence occurred after adjuvant therapy completed)	Sunitinib
		Sunitinib (recurrence occurred during adjuvant therapy)	BSC
Option 4	No	Imatinib	Sunitinib

*BSC* best supportive care

### Model structure

**Fig. 1:**
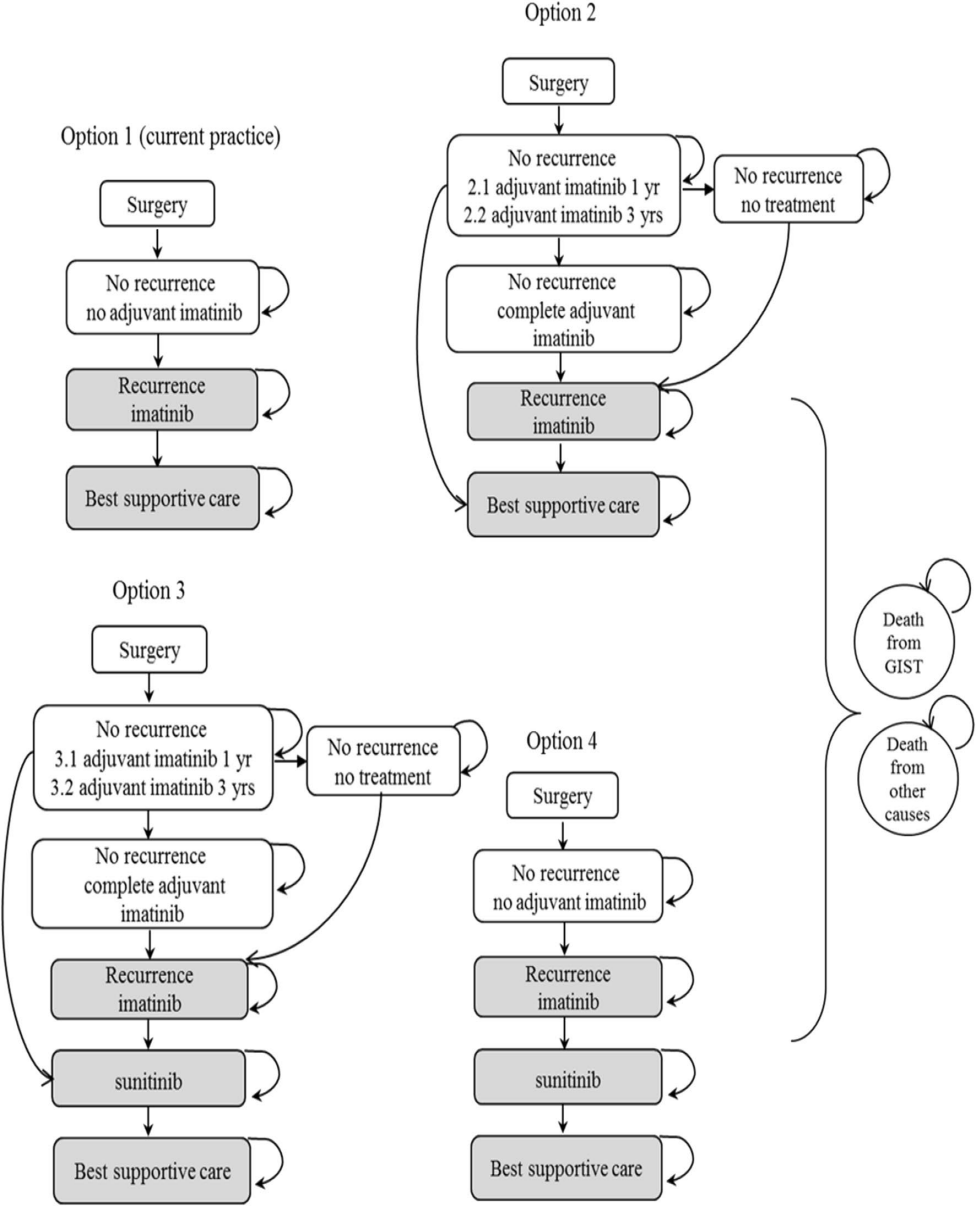
Markov model structure

The Markov model can be seen as illustrated in Fig. 1. There were three health states: (1) patients with no GIST recurrence; (2) patients with GIST recurrence; and (3) death from GIST and other causes. Four models were developed and based on the alternative treatment options.

The models started with patients aged 60 years old with no GIST recurrence after surgery. For options 1 and 4, no adjuvant imatinib therapy was applied. Patients with no disease recurrence could remain at the same state or move either to the recurrence state or death state. After patients entered the recurrence state (grey color), the states transitioned in accordance with each treatment option. For options 2 and 3, adjuvant imatinib was applied for 1 year or 3 years. Patients with adjuvant imatinib could either stop adjuvant treatment due to adverse events caused by the treatment or complete the treatment course. If recurrence occurred (grey color) after completing or during the adjuvant treatment, the states transitioned according to treatment options—similar to options 1 and 4.

For all health states, patients could remain in the same state or transition to the death state. The model assumed that recurrence was metastatic and not local as the literature showed that the proportion of metastatic recurrence after completed resections was higher [5].

### Clinical variables

Transitional probabilities of recurrence and death in patients with no adjuvant imatinib treatment were obtained from a retrospective chart review (Sarunporn Techasurungkul, Faculty of Medicine, Ramathibodi Hospital, Mahidol University, personal communication, November 20, 2014). A systematic review of randomized controlled trials (RCTs) published until 2014 was conducted to search for the hazard ratio of disease recurrence in patients receiving adjuvant imatinib treatment compared with no adjuvant imatinib treatment after surgery. Two studies were identified from the review: Joensuu et al. [7] investigated the efficacy of adjuvant imatinib treatment for 3 years compared with 1 year of adjuvant imatinib treatment in GIST patients with high risk of recurrence, while DeMatteo et al. [6] compared 1 year of adjuvant imatinib treatment with no adjuvant therapy. However, there was no study which compared 3 years of adjuvant imatinib treatment with no adjuvant therapy, and thus an indirect comparison analysis was performed from those two studies by using the ITC programme developed by the Canadian Agency for Drugs and Technologies in Health (CADTH) [11]. The values of the parameters are shown in Table 2.

**Table 2:** Parameters used in the model

Parameters	Distribution	Mean	SE	References
Probability of recurrence in patients with no adjuvant imatinib treatment (per month)				
Probability of recurrence at year 1	Beta	0.0205	0.0041	Chart review^a^
Probability of recurrence at year 3	Beta	0.0154	0.0031	
Probability of recurrence at year 5	Beta	0.0056	0.0011	
Probability of death from GIST in patients with no adjuvant imatinib treatment (per month)				
Probability of death at year 1	Beta	0.0017	0.0003	Chart review^a^
Probability of death at year 3	Beta	0.0031	0.0006	
Probability of death at year 5	Beta	0.0028	0.0006	
Probability of death at year 7	Beta	0.0020	0.0004	
Probability of death at year 9	Beta	0.0038	0.0008	
Hazard ratio (HR) of recurrence in patients receiving adjuvant imatinib treatment (compared with no adjuvant imatinib treatment)				
Adjuvant imatinib treatment for 1 year	Log normal	0.29	0.0995	[6]^b^
Adjuvant imatinib treatment for 3 years	Log normal	0.133	0.0543	Indirect comparison of [6] and [7]
Probability of progression during treatment (per month)				
Imatinib treatment	Beta	0.015	0.0038	[12]
Sunitinib treatment	Beta	0.012	0.0010	
Probability of death during treatment (per month)				
Imatinib treatment	Beta	0.0056	0.0009	[12]
Sunitinib treatment	Beta	0.0289	0.0087	
Best supportive care	Beta	0.0680	0.0093	
Probability of discontinuation of adjuvant imatinib treatment (per month)				
Adjuvant imatinib treatment for 1 year (1–6 months)	Beta	0.0136	0.0014	[17]
Adjuvant imatinib treatment for 1 year (7–12 months)	Beta	0.0009	0.0001	
Adjuvant imatinib treatment for 3 years (1–6 months)	Beta	0.0097	0.0001	
Adjuvant imatinib treatment for 3 years (7 months onwards)	Beta	0.0028	0.0003	
Direct medical costs (THB per month)				
Imatinib 400 mg/day	–	111,306	–	[14]
Sunitinib 50 mg/day (receive drug for 4 weeks, then stop for 2 weeks, and repeat a cycle)	–	82,173	–	
Costs for patients with no recurrence and no adjuvant imatinib treatment	Gamma	2758	308	Hospital database
Costs for patients with no recurrence and receiving adjuvant imatinib treatment^c^	Gamma	1477	573	
Costs for patients during recurrence and receiving imatinib treatment^c^	Gamma	421	38	[12]
Costs for patients during recurrence and receiving sunitinib treatment^c^	Gamma	714	150	
Costs for patients during recurrence and receiving best supportive care	Gamma	424	78	
Costs for treating adverse events from adjuvant imatinib treatment	Gamma	570	114	Expert opinion
Direct non-medical costs				
Travel costs (THB per visit)	Gamma	296	24	[15]
Food costs (THB per visit)	Gamma	109	11	
Opportunity cost of caregivers (THB per visit)	Gamma	99	37	
Number of visits for patients with no recurrence and no adjuvant imatinib treatment (visits per month)	Gamma	0.3	0.1	Hospital database
Number of visits for patients with no recurrence and receiving adjuvant imatinib treatment (visits per month)	Gamma	0.6	0.2	
Number of visits for patients during recurrence (visits per month)	–	1	–	[12]
Utility				
No recurrence and no adjuvant imatinib treatment	Beta	0.89	0.03	Interviewing patients
No recurrence and receiving adjuvant imatinib treatment	Beta	0.79	0.09	
Recurrence and receiving imatinib treatment	Beta	0.66	0.05	[12]
Recurrence and receiving sunitinib treatment	Beta	0.58	0.06	
Recurrence and receiving best supportive care	Beta	0.42	0.03	

^a^ Sarunporn Techasurungkul, Faculty of Medicine, Ramathibodi Hospital, Mahidol University, personal communication, November 20, 2014

^b^ The study population was patients with GIST of greater than 3 cm, so only a subgroup analysis of patients with GIST of greater than 10 cm was used to represent patients with high risk of recurrence

^c^Excluding drug costs (imatinib or sunitinib)

Since it was not clear that overall survival improved because of the adjuvant imatinib treatment, the improved duration of survival may have been due to treatments given after recurrence. Therefore, this study assumed that the probability of death during no recurrence was the same regardless of adjuvant therapy received. The probability of adjuvant therapy discontinuation was derived from literature review, and the probability of recurrence in this group was equal to patients with no adjuvant therapy. After recurrence, the transitional probabilities—including the probabilities of disease progression and death during the given treatment—were derived from a study by Mohara [12]. Additionally, mortality rates of the Thai population at each age were applied [13].

**Fig. 2:**
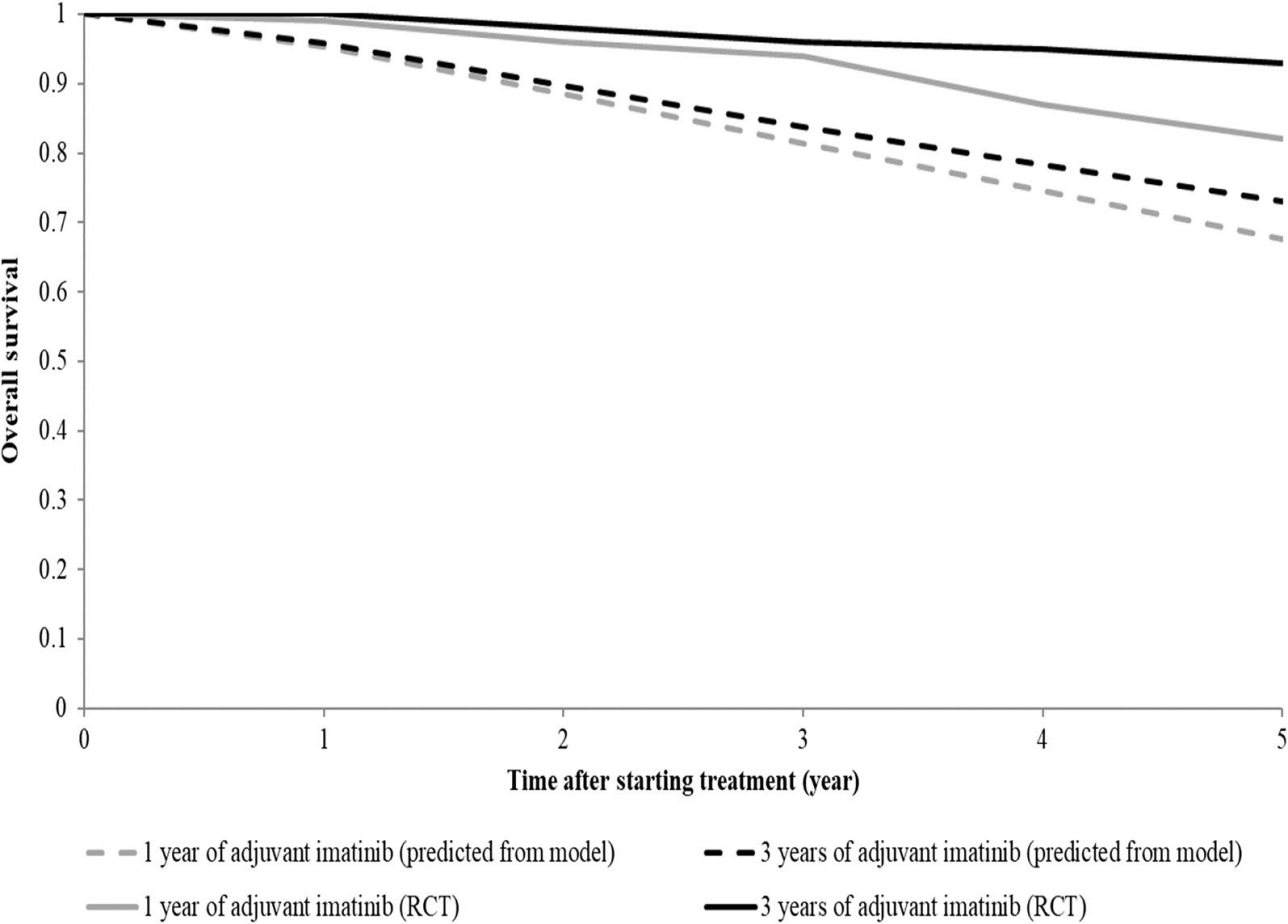
Overall survival of patients receiving adjuvant imatinib after surgery obtaining from the model and from the RCT

The model validation was performed by comparing survival estimation from the model and from an international RCT [7] as shown in Fig. 2. We found that our estimation provided similar results in terms of patient survival in the first 3 years. However, the model predicted a slightly higher mortality in the 4th and 5th year compared to that of RCT. It was because this study assumed, based on an agreement with local expert panel, that the treatment effect would be observed when patients were taking the medicine. This point is debatable, because there is no clear evidence in the current literature. Our findings, nonetheless, were acceptable to stakeholders in the consultation meeting conducted towards the end of the project, which was also a part of the model validation process. They indicated that this mortality estimation was representative of the real-world situation in Thailand.

### Costs

Direct medical and non-medical costs were included based on the societal perspective. Direct medical costs included the cost of drugs, cost of treating and monitoring, and cost of adverse event treatment. The reference prices of drugs were obtained from the Drug and Medical Supply Information Center (DMSIC), Ministry of Public Health. 400 mg of imatinib and 12.5 mg of sunitinib cost 3,659.40 Thai Baht (THB) per tablet and 1100.53 THB per capsule in 2014, respectively [14].

Resources used for treating and monitoring patients during no recurrence were collected from a review of medical charts in Songklanagarind Hospital during 2009–2014. Those were mostly from outpatient care, e.g. radiology services, laboratory services, outpatient services, and other drugs. Resources used for treating the adverse events caused by adjuvant imatinib treatment were obtained from expert opinion based on the adverse events that occurred from literature review [7]. Subsequently, costs were calculated according to those resources used and reference costs from the Thai standard costs list [15]. Moreover, direct non-medical costs such as the costs of travel, food, and opportunity cost of caregivers during no recurrence were estimated from the number of patients’ visits per month, obtained from a review of charts in Songklanagarind Hospital, and costs per visit were based on the Thai standard costs list.

Both direct medical and non-medical costs incurred during recurrence were retrieved from a Thai literature review [12]. All costs were adjusted to 2014 values by using the consumer price index (CPI).

### Health outcomes

Health outcomes in this study were life years (LYs) gained and quality-adjusted life years (QALYs) gained (multiplication of utility value and life years). The quality of life scores were collected by using the EQ-5D-3L questionnaire to interview 23 patients with high risk of GIST recurrence after surgery (5 patients who received adjuvant imatinib treatment and 18 patients who did not receive adjuvant imatinib treatment) at Ramathibodi and Songklanagarind Hospitals. To convert quality of life scores into utility values, Thai population coefficients were applied [16]. This study was approved by the Ethical Committees of Ramathibodi and Songklanagarind Hospitals.

### Data analysis

The incremental cost-effectiveness ratio (ICER) in THB per QALY gained was analyzed. The ICER was compared with the willingness-to-pay (WTP) threshold to determine whether each option was cost-effectiveness or not. In Thailand, the Subcommittee for Development of the NLEM and the Subcommittee for Development of the Health Benefit Package and Service Delivery, National Health Security Office (NHSO), set the WTP threshold at 160,000 THB per QALY gained.

A one-way sensitivity analysis was conducted to examine the effect of each individual parameter uncertainty, and the results were presented in a tornado diagram. A probabilistic sensitivity analysis (PSA) was also carried out to explore the effect of all parameter uncertainties using a Monte Carlo simulation approach. The simulation was run for 1000 times to yield the possible values for total costs, health outcomes, and ICER; these results are presented in cost-effectiveness acceptability curves. Additionally, if the current price of imatinib was found to be not cost-effective, a threshold sensitivity analysis was conducted to determine the price that would result in cost-effectiveness at the WTP threshold of 160,000 THB per QALY gained.

## Results

### Incremental cost-effectiveness ratio

**Fig. 3:**
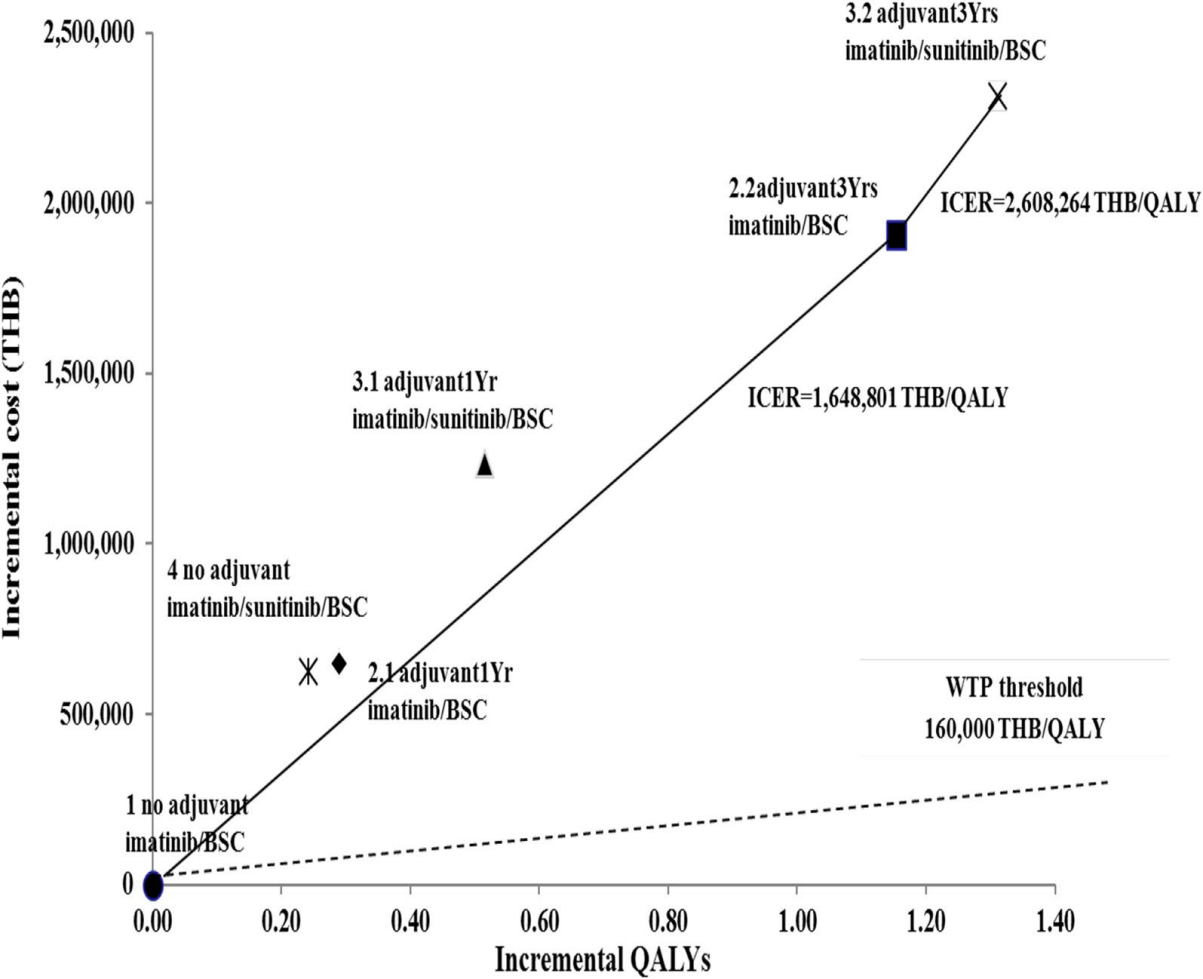
Cost-effectiveness plane and efficiency frontier

Table 3 shows the lifetime costs, outcomes, and ICER of alternative treatment options in GIST patients aged 60 years old and above with high risk of recurrence after surgery. It was found that option 3.2 yielded the highest lifetime cost (5.1 million THB) and outcomes (8.17 LYs and 6.65 QALYs). Meanwhile, option 1 had the lowest cost (2.7 million THB) and outcomes (6.72 LYs and 5.34 QALYs). All options were ranked in terms of cost —from the lowest to the highest—and the ICER of each option was calculated by comparing with that of the next option (e.g. option 4 comparing with option 1, option 2.1 comparing with option 4, etc.). Option 4, 2.1 and 3.1 were dominated by option 2.2 because they yielded less QALYs, but they resulted in a higher cost-effectiveness ratio compared to that of option 2.2. Therefore, option 2.2—patients who received adjuvant imatinib treatment for 3 years and continued to receive imatinib treatment and best supportive care depending on whether recurrence occurred after or during adjuvant therapy, respectively—was shown to be the best with the ICER of 1.6 million THB per QALY gained when compared to the current option (option 1). Option 3.2 was the next best option with an ICER of 2.6 million THB per QALY gained compared to option 2.2. Figure 3 shows the incremental cost and QALYs of each option comparing with the current option. The efficiency frontier was conducted by drawing a line between the better options where the slope between each option represented the ICER.

**Table 3:** Lifetime costs, outcomes, and ICERs of alternative treatment options

Treatment options	Cost (THB)	Life years		QALYs	ICER (THB per QALY gained)
		No discount	Discount		
Option 1 (current practice)	2,744,275	8.13	6.72	5.34	–
Option 4	3,368,809	8.57	7.05	5.58	Dominated by option 2.2
Option 2.1	3,393,388	8.56	7.01	5.63	Dominated by option 2.2
Option 3.1	3,979,869	8.97	7.33	5.86	Dominated by option 2.2
Option 2.2	4,648,080	9.87	7.95	6.50	1,648,801
Option 3.2	5,056,583	10.17	8.17	6.65	2,608,264

### Uncertainty analysis

**Fig. 4:**
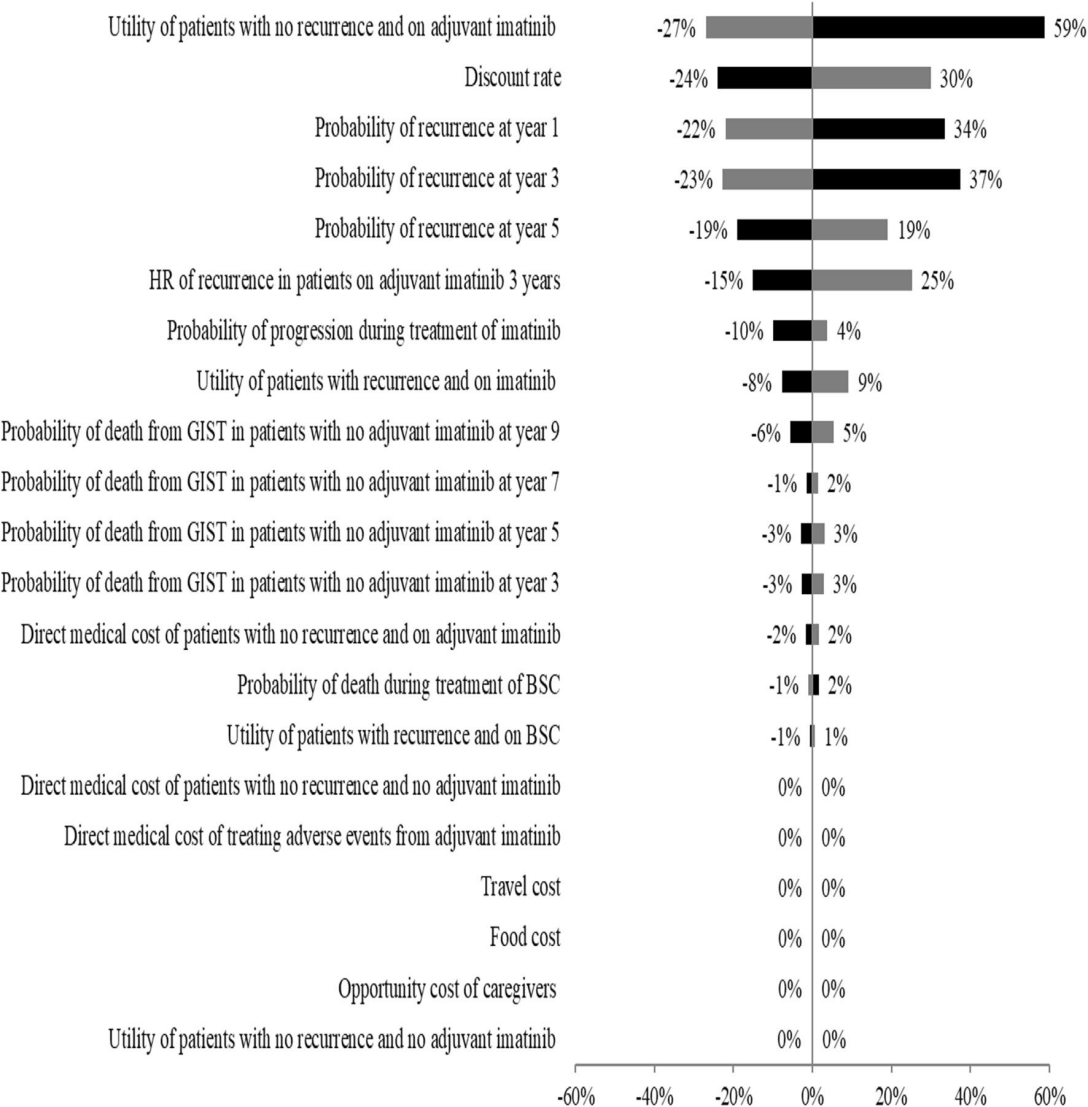
One-way sensitivity analysis

Figure 4 presents the one-way sensitivity analysis results of the best option (option 2.2) via a tornado diagram. These results showed the effect of varying each parameter within the 95% confidence interval (CI) on the ICER. The parameter that affected the ICER the most was the utility of patients with no recurrence and on adjuvant imatinib treatment, followed by discount rates of 0% and 6% per annum and the probability of recurrence.

**Fig. 5:**
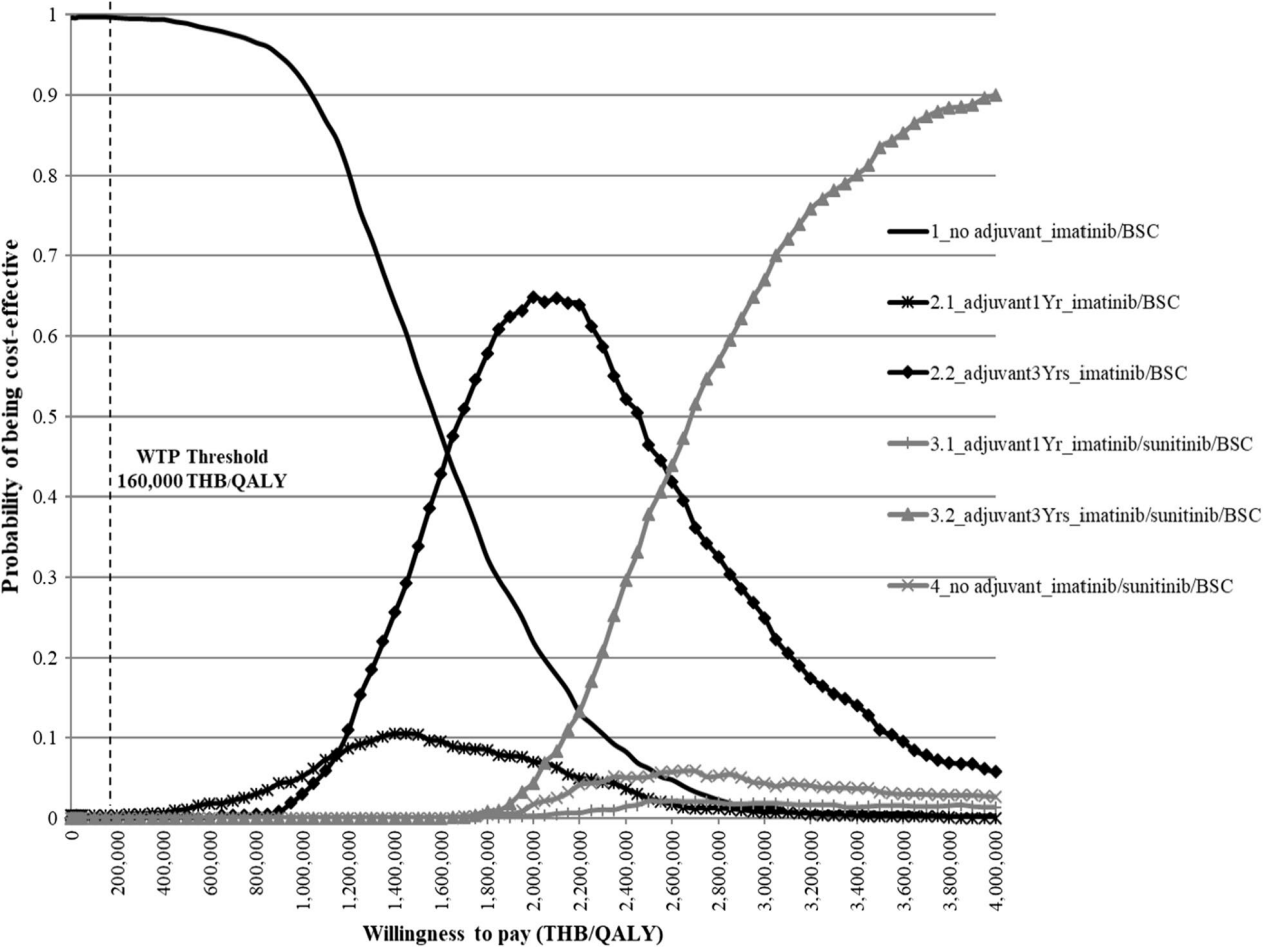
Cost-effectiveness acceptability curves

Figure 5 shows the PSA results, presented in cost-effectiveness acceptability curves. The results illustrated the probability of each treatment option being cost-effective at various WTP thresholds. It was found that at the WTP threshold of 160,000 THB per QALY gained, the probability of the current option being cost-effective was 100%, whereas the probability of other treatment options being cost-effective was 0%.

A threshold analysis was conducted to find the cost-effective price of providing adjuvant imatinib treatment at the WTP of 160,000 THB per QALY gained. Based on the option most likely to be cost-effective (option 2.2), adjuvant imatinib treatment would be cost-effective in the Thai context if the price of imatinib decreased from 3659 THB per 400 mg to 288 THB per 400 mg (a decrease of about 92%).

## Discussion

Patients with high risk of GIST recurrence who received adjuvant imatinib treatment after surgery would gain higher QALYs and yield higher lifetime costs as well when compared to no adjuvant therapy. The 3-year adjuvant imatinib treatment also produced higher QALYs and costs than 1 year of adjuvant imatinib treatment. Nevertheless, at the Thai WTP threshold of 160,000 THB per QALY gained, adjuvant imatinib treatment was not cost-effective in those patients compared to the current practice. A 3-year adjuvant imatinib treatment followed by imatinib treatment and best supportive care if recurrence occurred after or during adjuvant therapy, respectively, was the best option as it yielded the lowest ICER of 1,648,801 THB per QALY gained. This treatment option would be cost-effective if the price of imatinib 400 mg decreased from 3659 THB to 288 THB.

Studies on the cost-effectiveness of adjuvant imatinib treatment in GIST patients after surgery have been conducted in several countries. Two studies conducted in the United States and Netherlands were published in international journals [17, 18], while another three studies conducted in Canada, Scotland, and Greece were presented at the Congress of the European Society for Medical Oncology in 2012 [19–21]. All of those studies aimed to compare the cost-effectiveness of adjuvant imatinib treatment for 3 years with an adjuvant imatinib treatment for 1 year. Only the study conducted in Greece assessed other scenarios, in which one of those cost-effectiveness comparisons was between 3 years of adjuvant imatinib treatment versus no adjuvant therapy. The results of all the studies illustrated that adjuvant imatinib treatment for 3 years was cost-effective in their respective contexts when compared with either a 1 year of adjuvant imatinib treatment or no adjuvant therapy (only for the Greece study). The difference in conclusions between the other studies and this one was caused by differences in both interventions and comparators as this study assessed all treatment options based on the current clinical practice in Thailand. For example, the Greece study allowed patients to receive a high dose of imatinib before receiving sunitinib treatment when GIST recurrence occurred [20], but this study did not consider that treatment. This study was also the only one that compared treatment options after GIST recurrence, whereas other studies focused only on adjuvant imatinib treatment (treatment after GIST recurrence was the same between the 3-year and 1-year groups, and the proportion of patients receiving a particular treatment after GIST recurrence was based on expert opinion) [17, 18].

The sources of the parameters used in the model were also different, particularly for utility. Other studies derived the utilities of patients in each status from literature review or expert opinion; however, this study obtained them from interviewing Thai patients or from a Thai study that collected this data via interviews as well. Additionally, this study analyzed the results based on a societal perspective, whereas others used the payer’s or provider’s perspectives. On the other hand, similar to other studies, the results of this study indicated that 3 years of adjuvant imatinib treatment provided better health outcomes in terms of both LYs and QALYs, and yielded higher costs compared with 1 year of adjuvant imatinib treatment.

Several limitations were encountered in this study. Due to the fact that there was no study on the effectiveness of adjuvant imatinib treatment in Thai patients, the hazard ratios of recurrence obtained from international clinical trials for patients who received adjuvant imatinib treatment were applied with the probability of recurrence in Thai patients without adjuvant therapy in order to obtain the probability of recurrence in patients receiving adjuvant imatinib treatment that reflects the Thai population. However, those hazard ratios were applied only during the period that patients received adjuvant imatinib treatment, i.e. either for 1 or 3 years. This may, therefore, result in the underestimation of the effectiveness of adjuvant imatinib treatment.

The probabilities of recurrence and death of GIST patients without adjuvant imatinib after surgery were derived from medical records review, which could be bias due to the potential confounding factors that could not be taken out based on the study design—i.e. retrospective cohort study. It may the case that the patients without recurrence might not come back to see the doctors; hence, we might have overestimated the probability of recurrence due to the missing data from this group of patients. In contrast, the hospitals might not have medical records on patients who died at home, which could also lead us to underestimate the probability of dying. In summary, this limitation can both be positive and negative from the conclusion.

This study used two sources of data—i.e. primary data and secondary data, to obtain health utility of the patients. A primary data collection on health utility was conducted in 23 patients for only a health state of patients without GIST recurrence. Meanwhile, health utility of patients with GIST recurrence were obtained from another Thai study conducted by Mohara [12] which interviewed other 22 patients. This study employed data on health utilities from another Thai study rather than conducting primary data collection for all health states, because the target population in Mohara’s study and this study were the same –i.e. Thai patients with GIST. Mohara’s study obtained health utilities from patients with GIST recurrence using the same utility measure as ours—i.e. EQ5D, and the data was collected in recent years (in 2012). Moreover, Mohara’s study had already been used for informing policy decisions in Thailand. Since it was important to ensure our study’s consistency with the parameters used to inform policy decisions, we then borrowed data from that study to populate the model. It should be noted that the utility data of patients who received adjuvant imatinib treatment were collected from a small number of patients, because only a few patients had access to adjuvant imatinib treatment—it was expensive and could not be reimbursed. Nevertheless, the Thai HTA guidelines do not have recommendation on sample size calculation for health utility data collection. The guidelines suggest that the sample size should be as big as possible, given the time and resource constraint in conducting health economic evaluations. This recommendation is also in line with most other methodological guidelines that do not inform about sample size calculation for utility data measurement.

It was found from the one-way sensitivity analysis that the utility of patients who received adjuvant imatinib treatment, the probability of recurrence in high risk GIST after surgery, and the hazard ratio of recurrence in patients who received adjuvant imatinib treatment affected the ICER the most. However, this study extensively assessed the variability of these parameters in uncertainty analysis –in which we found almost 100% chance of making the right recommendation given the current ceiling threshold in Thailand. Therefore, pursuing another bigger study would provide no value added to policy decision making in Thailand.

## Conclusions

Adjuvant imatinib treatment in patients with high risk of GIST recurrence after surgery yielded better health outcomes, yet was not adequate enough to meet the cost-effective criteria in Thailand compared with no adjuvant therapy. The 3-year adjuvant imatinib treatment followed by imatinib treatment and best supportive care if recurrence occurred after or during adjuvant therapy, respectively, was the best option as it yielded the lowest ICER. Therefore, this treatment option should be applied if the price of imatinib can be negotiated down to an acceptable level.

## Abbreviations

GIST: gastrointestinal stromal tumour
GI: gastrointestinal
PDGFRA: platelet-derived growth factor receptor alpha
CT: computed tomography
MRI: magnetic resonance imaging
FDG-PET: fluorine-18-fluorodeoxyglucose positron emission tomography
RFS: recurrence-free survival
NCCN: National Comprehensive Cancer Network
ESMO: European Society for Medical Oncology
NIH: National Institutes of Health
AFIP: Armed Forces Institute of Pathology
NLEM: National List of Essential Medicines
BSC: best supportive care
RCTs: randomized controlled trials
CADTH: Canadian Agency for Drugs and Technologies in Health
DMSIC: Drug and Medical Supply Information Center
THB: Thai Baht
CPI: consumer price index
LYs: life years
QALYs: quality-adjusted life years
HR: hazard ratio
ICER: incremental cost-effectiveness ratio
WTP: willingness-to-pay
NHSO: National Health Security Office
PSA: probabilistic sensitivity analysis
CI: confidence interval
